# Supplementary material for: Empirical methods for controlling false positives and estimating confidence in ChIP-Seq peaks
Source: BMC Bioinformatics. 2008 Dec 5;9:523. doi: 10.1186/1471-2105-9-523 (PMC2628906; doi:10.1186/1471-2105-9-523)
Supplement: Additional File 1 — A variety of html documents from the USeq web site detailing the available applications, their best usage, output file type descriptions, command line menus, etc. [file 1471-2105-9-523-S1.zip › USeqUserGuides/JavaDoc.zip!/JavaDoc/util/xml/ParseXMLFile.html]

ParseXMLFile


|  |  |  |
| --- | --- | --- |
| **Overview** \| **Package** \| **Class** \| **Use** \| **Tree** \| **Deprecated** \| **Index** \| **Help** | |  |
| PREV CLASS   NEXT CLASS | **FRAMES**    **NO FRAMES**     **All Classes** |  |
| SUMMARY: NESTED \| FIELD \| CONSTR \| METHOD | DETAIL: FIELD \| CONSTR \| METHOD |  |


---


## util.xml Class ParseXMLFile

```
java.lang.Object
  util.xml.ParseXMLFile
```

---

``` public class ParseXMLFile extends java.lang.Object ```

This class represents short example how to parse XML file,
get XML nodes values and its values.  
  
It implements method to save XML document to XML file too

**Author:**
:   Martin Glogar

---

**Constructor Summary**

`ParseXMLFile()`
  
          Creates a new instance of ParseXMLFile


| **Method Summary** | |
| --- | --- |
| `static java.lang.String` | `getElementValue(org.w3c.dom.Node elem)`             Returns element value |
| `static void` | `main(java.lang.String[] args)`             Starts XML parsing example |
| `org.w3c.dom.Document` | `parseFile(java.lang.String fileName)`             Parses XML file and returns XML document. |
| `boolean` | `saveXMLDocument(java.lang.String fileName, org.w3c.dom.Document doc)`             Saves XML Document into XML file. |
| `void` | `writeDocumentToOutput(org.w3c.dom.Node node, int indent)`             Writes node and all child nodes into System.out |
| `void` | `writeGenesToOutput(org.w3c.dom.Node node)`             Writes node and all child nodes into System.out |

**Methods inherited from class java.lang.Object**

`equals, getClass, hashCode, notify, notifyAll, toString, wait, wait, wait`

**Constructor Detail**

### ParseXMLFile

```
public ParseXMLFile()
```

:   Creates a new instance of ParseXMLFile


**Method Detail**

### getElementValue

```
public static final java.lang.String getElementValue(org.w3c.dom.Node elem)
```

:   Returns element value

    :   **Parameters:**: `elem` - element (it is XML tag) **Returns:**: Element value otherwise empty String

---


### writeDocumentToOutput

```
public void writeDocumentToOutput(org.w3c.dom.Node node,
                                  int indent)
```

:   Writes node and all child nodes into System.out

    :   **Parameters:**: `node` - XML node from from XML tree wrom which will output statement start: `indent` - number of spaces used to indent output

---


### writeGenesToOutput

```
public void writeGenesToOutput(org.w3c.dom.Node node)
```

:   Writes node and all child nodes into System.out

    :   **Parameters:**: `node` - XML node from from XML tree wrom which will output statement start: `indent` - number of spaces used to indent output

---


### saveXMLDocument

```
public boolean saveXMLDocument(java.lang.String fileName,
                               org.w3c.dom.Document doc)
```

:   Saves XML Document into XML file.

    :   **Parameters:**: `fileName` - XML file name: `doc` - XML document to save **Returns:**: **true** if method success **false** otherwise

---


### parseFile

```
public org.w3c.dom.Document parseFile(java.lang.String fileName)
```

:   Parses XML file and returns XML document.

    :   **Parameters:**: `fileName` - XML file to parse **Returns:**: XML document or **null** if error occured

---


### main

```
public static void main(java.lang.String[] args)
```

:   Starts XML parsing example

    :   **Parameters:**: `args` - the command line arguments


---


|  |  |  |
| --- | --- | --- |
| **Overview** \| **Package** \| **Class** \| **Use** \| **Tree** \| **Deprecated** \| **Index** \| **Help** | |  |
| PREV CLASS   NEXT CLASS | **FRAMES**    **NO FRAMES**     **All Classes** |  |
| SUMMARY: NESTED \| FIELD \| CONSTR \| METHOD | DETAIL: FIELD \| CONSTR \| METHOD |  |


---
